# Hyperbaric Oxygen Treatment at Various Stages following Chronic Constriction Injury Produces Different Antinociceptive Effects via Regulation of P2X_4_R Expression and Apoptosis

**DOI:** 10.1371/journal.pone.0120122

**Published:** 2015-03-19

**Authors:** Bai-Song Zhao, Xing-Rong Song, Pei-Ying Hu, Ling-Xin Meng, Yong-Hong Tan, Ying-Jun She, Yuan-Yuan Ding

**Affiliations:** 1 Department of Anesthesiology, Guangzhou Women and Children’s Medical Center, 510623, Guangzhou, China; 2 Department of Pain, The Shengjing Hospital of China Medical University, 110004, Shenyang, China; University of Sao Paulo, BRAZIL

## Abstract

**Purpose:**

The aims of this study were to investigate the effect of hyperbaric oxygen (HBO) treatment at various stages following chronic constriction injury (CCI) and to explore the underlying mechanisms of HBO treatment.

**Methods:**

Forty adult male Sprague—Dawley rats were randomly assigned to five groups (n = 8 for each group): the sham group, CCI group, HBO1 group, HBO2 group, and HBO3 group. Neuropathic pain was induced by CCI of the sciatic nerve. HBO treatment began on postoperative days 1, 6, and 11 and continued for 5 days. The mechanical withdrawal threshold and thermal withdrawal latency were tested on preoperative day 3 and postoperative days 1, 3, 5, 7, 10, 14, and 21. The expression of P2X_4_R was determined by immunohistochemistry and western blot analysis. Cell apoptosis was measured using TUNEL staining. The expression of caspase 3 was measured using reverse transcription polymerase chain reaction (RT-PCR). Electron microscopy was used to determine the ultrastructural changes.

**Results:**

Early HBO treatment beginning on postoperative day 1 produced a persistent antinociceptive effect and inhibited the CCI-induced increase in the expression of P2X_4_R without changing CCI-induced apoptosis. In contrast, late HBO treatment beginning on postoperative day 11 produced a persistent antinociceptive effect and inhibited CCI-induced apoptosis and upregulation of caspase-3 without changing the expression of P2X_4_R. In addition, late HBO treatment reduced CCI-induced ultrastructural damage. However, HBO treatment beginning on postoperative day 6 produced a transient antinociceptive effect without changing the expression of P2X_4_R or CCI-induced apoptosis.

**Conclusion:**

HBO treatment at various stages following CCI can produce antinociceptive effects via different mechanisms. Early HBO treatment is associated with inhibition of P2X_4_R expression, and late HBO treatment is associated with inhibition of cell apoptosis.

## Introduction

Neuropathic pain is commonly caused by central or peripheral nerve injury, characterized by allodynia, hyperalgesia, spontaneous pain, and paraesthesia [1]. Although the mechanisms underlying neuropathic pain are complex and unclearly understood, it is generally believed that neuropathic pain is the result of direct or indirect injury of a sensory nerve [2,3]. As one of the most intractable types of chronic pain, neuropathic pain causes a great burden to society and patients; in addition, it causes physiological, psychological, and social problems that impair the quality of life of patients [4,5]. Several systemic retrospective studies have shown that patients with neuropathic pain have a lower quality of life than those with chronic diseases such as cancer, diabetes, chronic heart failure, and stroke [6,7]. In addition, epidemiological studies have shown that the incidence of neuropathic pain is 6–8% in the general population, including 22% of patients with chronic pain and 74% of patients with moderate or severe pain [8–10]. Therefore, neuropathic pain is a severe clinical problem that challenges clinicians.

Hyperbaric oxygen (HBO) treatment, in which patients receive 100% oxygen at a pressure higher than atmospheric pressure, has been widely used in the clinic. HBO treatment has some advantages such as being a noninvasive procedure, causing minimal side effects, and being easy to carry out; thus, it is willingly accepted by patients. HBO treatment has been used for the treatment of neurological diseases such as stroke, spinal cord injury, and cerebral ischemia [11–13]. Several lines of evidence have shown that HBO treatment exerts neuroprotective effects via mechanisms including inhibition of inflammation, reduction of hypoxia, and improvement of microcirculation in the nervous system [14–16].

HBO treatment has been used to treat neuropathic painin rats following acute thoracic spinal cord injury [17]. Gu et al. have reported that HBO treatment effectively attenuated neuropathic pain in both an animal model of neuropathic pain and in patients with idiopathic trigeminal neuralgia [18]. Several mechanisms such as neural protection, anti-inflammation, and inhibition of nerve injury-induced changes in neural activity may contribute to the analgesic effect of HBO therapy [18]. Gibbons et al. have found that HBO treatment reduced sciatic nerve crush-induced neuropathic pain in rats possibly via activation of opioid receptors [19]. In addition, Li et al. have found that HBO therapy reduced chronic constrictive injury-induced neuropathic pain and reduced the production of tumor necrosis factor-α [20]. Furthermore, we have found previously that the antinociceptive effect of HBO treatment is associated with astrocyte inhibition and anti-inflammation [21]. Therefore, although the antinociceptive mechanisms of HBO treatment are not well understood, it appears that multiple different mechanisms contribute to the antinociceptive effects of HBO treatment. The identification of the antinociceptive mechanisms of HBO treatment may reveal a novel strategy for the treatment of neuropathic pain.

Neuropathic pain at different stages has different underlying mechanisms, and elucidation of the mechanisms underlying neuropathic pain at different stages may be useful for developing [22] therapeutic targets for drug therapy used at a specific stage of neuropathic pain. We have found previously that HBO treatment at 2 and 2.5 atm absolute pressure (ATA) beginning on postoperative day 1 produces a long-acting antinociceptive response in rats with chronic constriction injury (CCI) [21]. However, the antinociceptive effect of HBO treatment beginning at various stages following CCI and its underlying mechanisms remain unknown. In the present study, we aimed to investigate the antinociceptive effect of HBO treatment beginning on postoperative days 1, 6, and 11. It has been reported that P2X_4_R, a ligand-gated ion channel activated by ATP, is involved in the generation and maintenance of neuropathic pain [23] and that neuronal apoptosis is associated with the time course of neuropathic pain [24]. Therefore, we further investigated whether the expression of P2X_4_R and neuronal apoptosis are involved in the antinociceptive mechanisms of HBO treatment.

## Materials and Methods

### Animals

This study was carried out in strict accordance with the recommendations in the Guide for the Care and Use of Laboratory Animals of the National Institutes of Health. The protocol was approved by the Institutional Animal Ethics Committee of China Medical University. All surgery was performed under sodium pentobarbital anesthesia, and all efforts were made to minimize suffering.Forty adult male Sprague—Dawley rats (8–10 weeks old, weighing 250–280 g) were used in this study. The animals were housed individually in plastic boxes at 23–25°C with standard chow and water available *ad libitum*. Forty rats were randomly assigned to five groups (n = 8 for each group): the sham group, CCI group, HBO1 group (HBO treatment for 5 days beginning on postoperative day 1), HBO2 group (HBO treatment for 5 days beginning on postoperative day 6), and HBO3 group (HBO treatment for 5 days beginning on postoperative day 11).

### Induction of neuropathic pain

The CCI model of the sciatic nerve was used to create neuropathic pain as described previously [21,25]. Briefly, rats were anesthetized by intraperitoneal injection of sodium pentobarbital (40 mg/kg). The left biceps femoris of each rat was bluntly dissected at the mid-thigh level to expose the sciatic nerve. Four 4–0 chromic catgut sutures were loosely tied around the sciatic nerve at 1-mm intervals immediately proximal to the trifurcation. The wound was then sutured in layers. For the sham group, an identical dissection was performed, but the sciatic nerve was not ligated.

### Hyperbaric oxygen treatment

The cylindrical HBO treatment chamber (DS400-IV, Weifang Huaxin Oxygen Industry Co., Ltd., Shandong, China) was precoated with soda lime on the bottom to minimize water vapor and CO_2_ accumulation. The chamber was ventilated with 10% oxygen for 10 min. After a rat was placed in the chamber, the pressure was increased at a rate of 0.1 ATA/min to the desired pressure (2.0 ATA) and maintained for 60 min. The rats were allowed to breathe spontaneously during the HBO treatment. The chamber was then decompressed to normal room pressure at a rate of 0.1ATA/min. For rats in the HBO1, HBO2, and HBO3 groups, HBO treatment began on postoperative days 1, 6, and 11, respectively. All rats received HBO treatment once a day for 5 days. Rats in the sham and CCI groups were placed inside the chamber without HBO treatment.

### Behavioral tests

Mechanical withdrawal threshold (MWT) and thermal withdrawal latency (TWL) tests were performed on preoperative day 3 and postoperative days 1, 3, 5, 7, 10, 14, and 21. Each animal was placed in a Plexiglas chamber and habituated for 1 h prior to each test session before and after HBO treatment.

The MWT test was carried out to assess the response of the paw to a mechanical stimulus. The rats were placed in a Plexiglas chamber, and the MWT test was performed by stimulating the plantar surface of the left hind paw using von Frey filaments (Stoelting Company, USA). Each von Frey filament was held for approximate 3–5 s. Each trial started with the application of a 0.6-g von Frey force following the up-and-down procedure. A positive response was defined as a quick withdrawal of the hind paw upon stimulation. When a positive response occurred, a filament with a lower force was applied. If a negative response occurred, a filament with a greater force was applied. This protocol was continued until the least force that caused withdrawal was identified. The cut off value was 15 g. The paw threshold test was performed ten times, and the paw withdrawal threshold was defined as the von Frey force that caused 50% withdrawal.

To examine TWL, a BME-410C full-automatic plantar analgesia tester (Youer Equipment Scientific Co., Ltd., Shanghai, China) was used to measure the sensitivity of the paw to thermal stimuli. The thermal withdrawal latency test was performed by placing the rats on the surface of a 3-mm-thick glass plate that was covered with the same Plexiglas chamber. The radiant heat source was positioned at a fixed distance below the glass plate. Heat stimuli were directed at the exposure site on the left hind paw. The TWL was defined as the elapsed time (in seconds) to withdraw the paw from the heat source. Each test session included the delivery of five thermal stimuli at 5-min intervals, and the mean latency was used. A cut-off time of 30 s was set to avoid tissue damage.

### Tissue preparation

After completion of the behavioral tests on postoperative day 21, the rats were anesthetized by intraperitoneal injection of 10% chloral hydrate (300 mg/kg). The rats were transcardially perfused with 200 mL of normal saline. The spinal cord between the L4 and L6 segments was removed. The tissues were stored at -80°C and used for reverse transcription polymerase chain reaction (RT-PCR) (n = 2 rats per group) and western blot (n = 2 rats per group). The tissues were post-fixed in 4% paraformaldehyde for 24 h, dehydrated in 30% sucrose in PBS at 4°C for 24 h, and embedded in paraffin. These tissue blocks were used for immunohistochemistry (n = 2 rats per group) and TUNEL staining (n = 2 rats per group).

### Immunohistochemistry

Spinal cord sections (5 μm thick) were obtained from formalin-fixed and paraffin-embedded tissue blocks. Endogenous peroxidase activity was blocked by 3% H_2_O_2_ at room temperature for 15 min. Sections were incubated in 10% normal goat serum for 10 min to block nonspecific protein binding sites. Sections were then incubated with primary antibodies against P2X_4_R (1:100 dilution, Santa Cruz Biotechnology, USA) overnight at 4°C. After the primary antibody was washed off, the sections were incubated with goat anti-rabbit biotin-conjugated secondary antibodies (1:1000 dilution, Santa Cruz Biotechnology, USA) for 1 h at 37°C. The sections were then incubated with streptavidin horseradish peroxidase for 30 min at 37°C. The substrate 3,3′-diaminobenzidine (DAB) was applied to the section for 5 min, and the sections were then counterstained with hematoxylin. Sections in which primary antibodies were omitted were used as negative controls. The immunostained sections were examined under a light microscope. Five sections were selected from each rat. For each section, five fields were randomly selected in the left dorsal horn at a high-power field (×400 magnification). For each section, stained cells were counted, and the percentage of P2X_4_R-postive cells was calculated by dividing the number of P2X_4_R-postive cells by the total number of cells. The average of five sections was used for comparison among groups.

### Western blot

Spinal cord tissues were homogenized on ice in lysis buffer. Protein concentrations were determined using the BCA method. The proteins were resolved by sodium dodecyl sulfate—polyacrylamide gel electrophoresis and transferred onto polyvinylidene fluoride membranes by electroblotting. The membranes were incubated with primary antibodies against P2X_4_R (dilution 1:1000, Santa Cruz Biotechnology, USA) at 4°C overnight. β-Actin was used as a loading control. The membranes were then incubated with alkaline phosphatase-linked mouse anti-rabbit secondary antibodies (dilution 1:2000, Santa Cruz Biotechnology, USA) at room temperature for 2 h. The bands were visualized using a chemiluminescence detection system and analyzed with ScionImage software. The expression of P2X_4_R was normalized to that of β-actin.

### TUNEL staining

The tissue blocks were postfixed in 4% paraformaldehyde for 24 h and embedded in paraffin. Serial sections (5 μm) were obtained from the paraffin-embedded tissue blocks. The sections were deparaffinized in xylene and dehydrated with serial dilutions of alcohol followed by a wash in distilled water. After treatment with 3% H_2_O_2_ for 10 min at room temperature, the sections were incubated with proteinase K (1:200 in Tris-buffered saline (TBS)) for 15 min at 37°C. TUNEL staining was performed using a BioVision kit (BioVision Co., USA). The sections were then incubated with TdT and dUTP-digoxigenin in a humidified chamber at 37°C for 2 h, followed by three washes in TBS. The sections were then incubated with streptavidin-biotin complex (SABC, Sigma-Aldrich Co. Ltd., USA) at 37°C for 60 min and colorized with DAB for 10 min. The sections were counterstained with hematoxylin, then examined under a light microscope, and analyzed with Image-pro plus 6.0 software. Three sections were selected from each rat in each group. For each section, five fields were randomly selected in the injured area at a high-power field (×400 magnification). For each section, the stained cells were counted, and the apoptotic ratio was calculated according to the following formula: apoptotic ratio = the number of TUNEL-positive cells / the total number of cells. The average of three sections was used for comparison among groups.

### RT-PCR

Total RNA was isolated from the spinal cord by using the Trizol reagent (Invitrogen, USA), according to manufacturer’s protocol. RNA was reverse-transcribed into complementary DNA (cDNA) using the TaKaRa reverse transcription kit (TaKaRa Biotechnology Co., Dalian, China), according to the manufacturer’s instructions. RT-PCR was performed using a MyCycler PCR machine (Bio-Rad, USA). Primers for caspase 3 were 5′-AAGAAGACCATAGCAAAAGGAG-3′ (forward) and 5′-CACAAAGTGACTGGATGAACC-3′ (size, 349 bp). β-Actin was used as an internal control. The reaction conditions were as follows: 94°C for 3 min; 94°C for 30 s, 62°C for 30 s, and 72°Cfor 1 min for 35 cycles; and 72°C for 8 min. PCR products were resolved by 1.5% agarose gel electrophoresis containing ethidium bromide. Scion image software was used to quantify the mRNA expression levels. Relative caspase 3 expression was normalized to β-actin expression.

### Electron microscopy

To determine the ultrastructure of the spinal cord, 1-mm tissue pieces in the dorsal horn of the spinal cord (n = 2 rats per group) were removed and fixed with 2.5% glutaraldehyde. After three 10-min washes with 0.1 M PBS, they were postfixed in 1% OsO_4_ for 1 h, dehydrated in a graded series of acetone solutions, and then embedded in a 1:1 mixture of Epon812 and acetone for 2–3 h, followed by embedment in Epon812 for 2 h. Ultrathin sections were cut, stained with lead citrate and uranyl acetate, and viewed and imaged with an electron microscope.

### Statistical analysis

Analyses were performed using SPSS 17.0 (SPSS Inc., Chicago, IL, USA). Numerical data are presented as the mean and standard deviation. One-way analysis of variance was used to compare differences among groups, followed by the least significant difference test. Statistical significance was considered as *p*< 0.05.

## Results

### HBO treatment at various stages following CCI produces different antinociceptive effects

**Fig 1:**
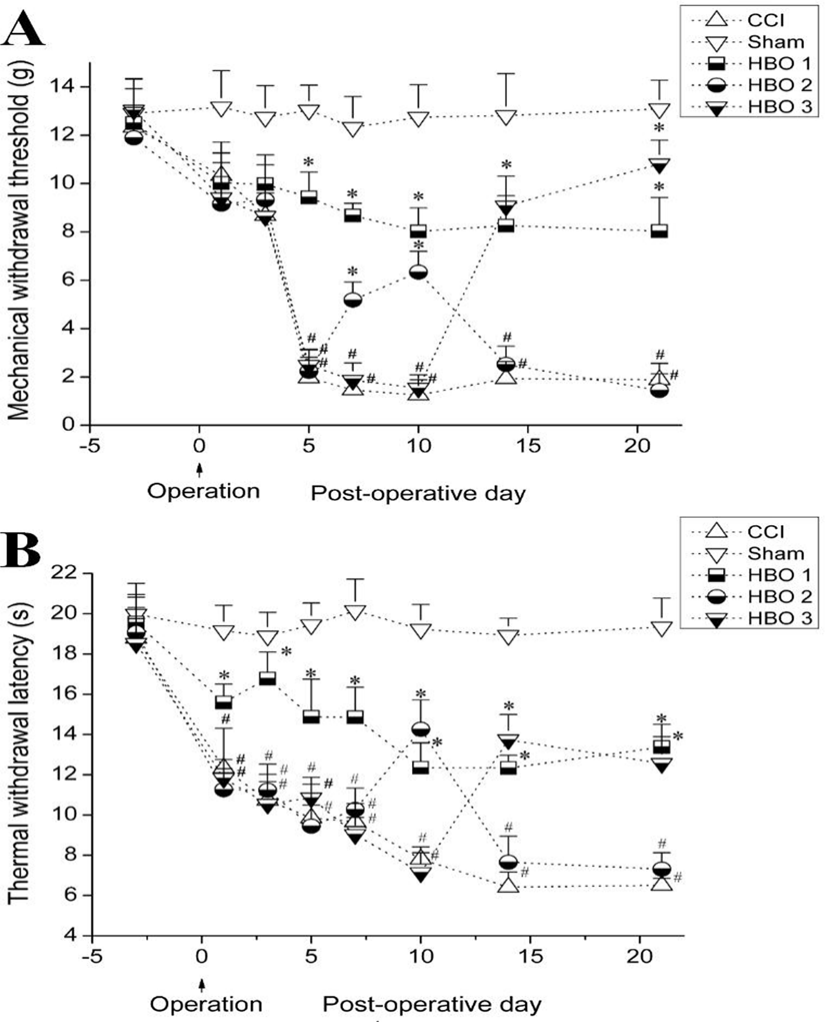
Effects of HBO treatment at various stages following CCI on the MWT (A) and TWL (B) in rats in the sham, CCI, HBO1 (HBO treatment beginning on postoperative day 1), HBO2 (HBO treatment beginning on postoperative day 6), and HBO3 (HBO treatment beginning on postoperative day 11) groups. #p < 0.05 vs. sham, *p < 0.05 vs. CCI.

There were no significant differences in the preoperative MWT and TWL among groups. Compared with the sham group, the MWT and TWL significantly decreased on postoperative days 3–21 in the CCI group (Fig. 1), suggesting that CCI induced persistent mechanical and thermal hyperalgesia in rats. Compared with the CCI group, the MWT and TWL were significantly higher on postoperative days 5–21 in the HBO1 group, suggesting that early HBO treatment beginning on postoperative day 1 produced a persistent antinociceptive effect. However, a significant increase in the MWT and TWL was only observed on postoperative days 7 and 10, but not on postoperative days 14 and 21, suggesting that late HBO treatment beginning on postoperative day 6 produced a transient antinociceptive effect. Furthermore, the MWT and TWL were significantly higher on postoperative days 14–21 in the HBO3 group compared with the CCI group, suggesting that late HBO treatment beginning on postoperative 11 produced a persistent antinociceptive effect (Fig. 1). These findings suggested that HBO treatment beginning at various stages following CCI produced different antinociceptive effects.

### Early HBO treatment reduces the CCI-induced increase in the expression of P2X4R in the spinal cord

**Fig 2:**
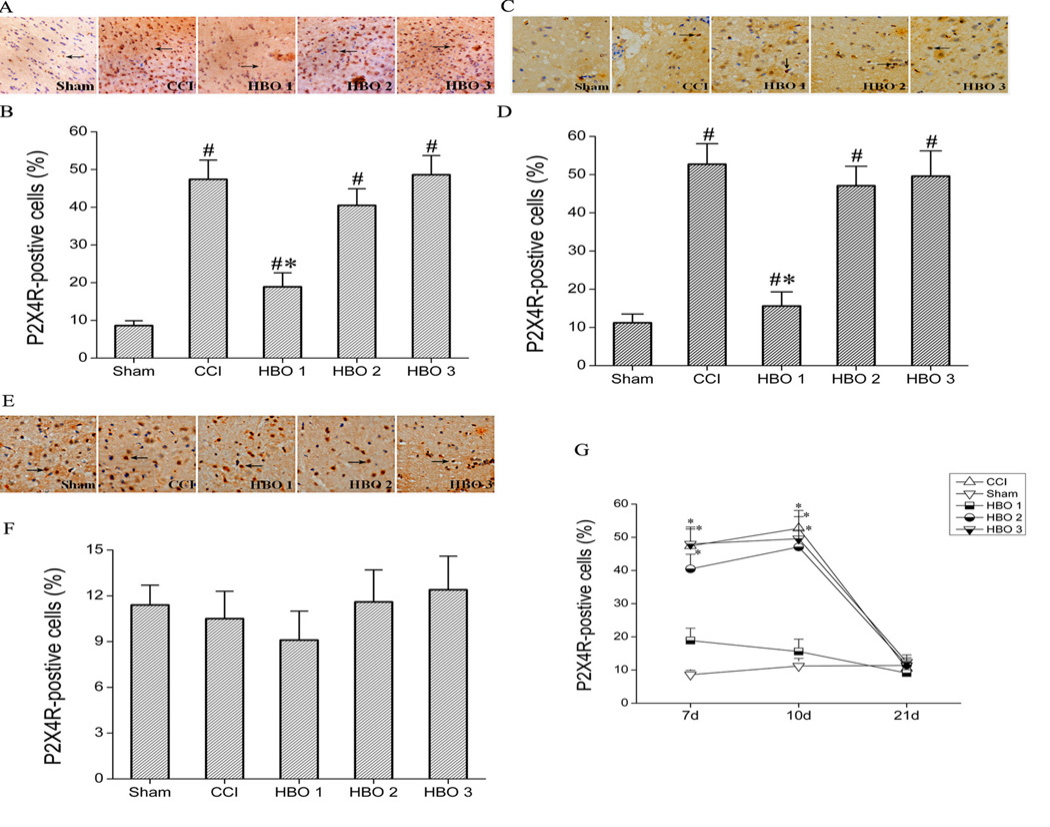
Immunohistochemical photomicrographs and bar graph showing the expression of P2X_4_R in the spinal cord of rats on postoperative days 7 (A–B), 10 (C–D), and 21 (E–F) in the sham, CCI, HBO1, HBO2, and HBO3 groups. P2X_4_R immunopositive cells are indicated by arrows. Magnification: ×400. G. The time course of the expression of P2X_4_R on postoperative days 7, 10, and 21 in the sham, CCI, HBO1, HBO2, and HBO3 groups. #p < 0.05 vs. sham, *p < 0.05 vs. CCI.

Next, we examined the expression of P2X_4_R in the spinal cord of rats after HBO treatment at various stages following CCI using immunohistochemistry. On postoperative day 7, compared with the sham group, CCI significantly increased the expression of P2X_4_R by approximately 5.5-fold in the spinal cord (Fig. 2A, B, G). The expression of P2X_4_R continued to increase until 10 days after CCI and declined to almost control levels by postoperative day 21 (Fig. 2A-G). The CCI-induced increase in the expression of P2X_4_R on postoperative days 7 and 10 was significantly inhibited by early HBO treatment beginning on postoperative day 1 (HBO1 group), but not by HBO treatment on postoperative days 6 and 11 (HBO2 and HBO3 groups) (Fig. 2A-D, G). On postoperative day 21, the expression of P2X_4_R in all five groups returned to almost control levels, and there was no significant difference in the expression of P2X_4_R among the five groups (Fig. 2E-G).

### Late HBO treatment inhibits cell apoptosis and downregulates the expression of caspase 3

**Fig 3:**
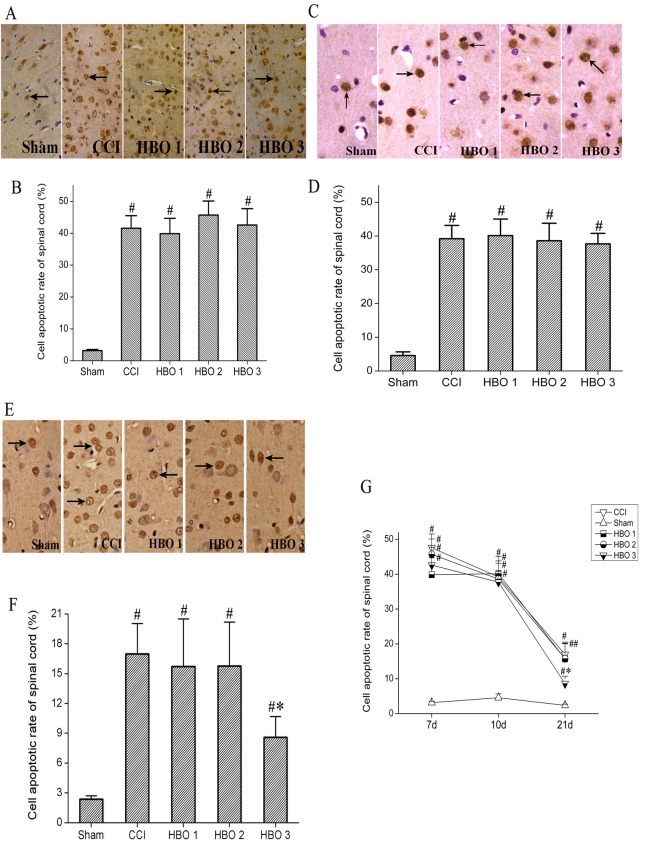
TUNEL staining showing cell apoptosis in the spinal cord of rats on postoperative days 7 (A–B), 10 (C–D), and 21 (E–F) in the sham, CCI, HBO1, HBO2, and HBO3 groups. Apoptosis was expressed as the percentage of the number of TUNEL-positive cells to the total number of cells. G. The time course of cell apoptosis on postoperative days 7, 10, and 21 in the sham, CCI, HBO1, HBO2, and HBO3 groups. #p < 0.05 vs. sham, *p < 0.05 vs. CCI.

**Fig 4:**
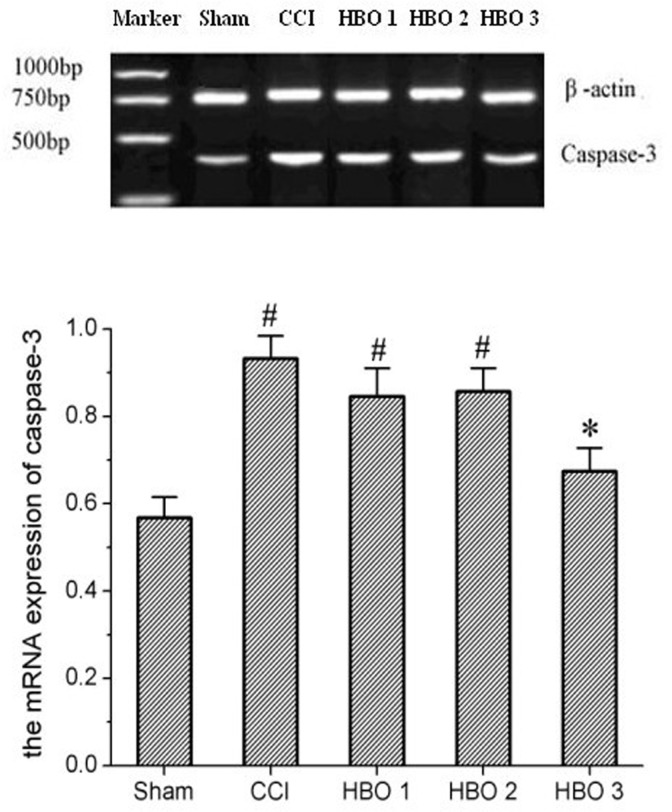
RT-PCR results showing the expression of caspase 3 on postoperative day 21 in the sham, CCI, HBO1, HBO2, and HBO3 groups. #p < 0.05 vs. sham, *p < 0.05 vs. CCI.

On postoperative day 7, compared with the sham group, the number of apoptotic cells was significantly increased by approximately 7-fold in the CCI group (Fig. 3A, B, G). Cell apoptosis declined at 10 days after CCI and remained higher than the control level on postoperative day 21 (Fig. 3A-G). On postoperative days 7 and 11, early and middleHBO treatment (HBO1 and HBO2 groups) did not prevent CCI-induced cell apoptosis (p > 0.05, Fig. 3A-D, G). However, on postoperative day 21, late HBO treatment beginning on postoperative day 11, but not early and middle HBO treatment, significantly inhibited CCI-induced cell apoptosis by approximately 2-fold (p < 0.05, Fig. 3E-G). In addition, late HBO treatment, but not early or middle HBO treatment, significantly inhibited the CCI-induced increase in the expression of caspase 3 (Fig. 4).

### Late HBO treatment reduces CCI-induced ultrastructural damage

**Fig 5:**
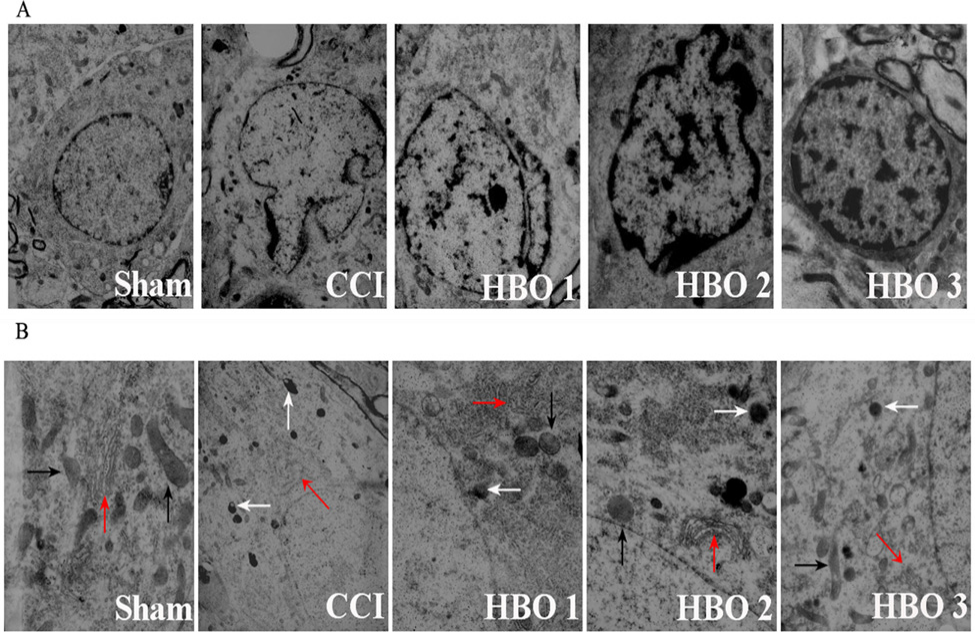
Electron micrographs showing the ultrastructure of spinal cord neurons in rats in the sham, CCI, HBO1, HBO2, and HBO3 groups. A. Representative electron micrographs showing the nucleus. Magnification: ×6000. B. Representative electron micrographs showing mitochondria (black arrows), rough endoplasmic reticulum (red arrows), and lysosomes (white arrows). Magnification: ×12,000.

In the sham group, the neurons had round nuclei with a clear nuclear membrane and nucleoli. Chromatin was evenly distributed inside the nuclei (Fig. 5A). The cytoplasm was rich in ribosomes, rough endoplasmic reticulum, mitochondria, and Golgi complexes (Fig. 5B). In the CCI groups, the neurons had round nuclei with an unclear nuclear membrane. Ruptured cytoplasmic membranes and accumulated heterochromatin in the periphery were observed (Fig. 5A). The number of lysosomes in the cytoplasm was decreased, and the outer cristae of the mitochondria were unclear. An increase in the number of lysosomes, expansion of the endoplasmic reticulum, and swelling and vacuolation of mitochondria were noted(Fig. 5B). Early and middle HBO treatment did not reduce the CCI-induced ultrastructural damage. However, late HBO treatment reduced the CCI-induced damage, as indicated by the findings of a clear nuclear membrane (Fig. 5A), more ribosomes and rough endoplasmic reticulum, a clear outer membrane of the mitochondria, and fewer vacuolated mitochondria (Fig. 5B).

## Discussion

We have previously found that HBO treatment beginning on postoperative day 1 alleviates neuropathic pain in CCI rats [21]. In the present study, we further investigated the antinociceptive effect of HBO treatment beginning at various stages following CCI. HBO treatment beginning on postoperative day 1 produced a persistent antinociceptive effect that was associated with inhibition of the CCI-induced increase in the expression of P2X_4_R in the spinal cord. In contrast, HBO treatment beginning on postoperative day 11 produced a persistent antinociceptive effect that was associated with inhibition of CCI-induced neuronal apoptosis. However, HBO treatment beginning on postoperative day 6 produced a transient antinociceptive response only during the period of HBO treatment, and the antinociceptive effect disappeared after HBO treatment was stopped. Thus, our study suggests that HBO treatment at various stages following CCI produces different antinociceptive responses through different mechanisms.

In the present study, we investigated the expression of P2X_4_R in the spinal cord in CCI rats receiving HBO treatment at various stages following CCI. It has been reported that the expression of P2X_4_R in the spinal cord and hippocampus starts to increase soon after neuropathic pain[26,27]. In the present study, we found that the MWT and TWL started to decline on postoperative day 3, reached the nadir on postoperative day 7, and was maintained for 14 days. Consistent with the early changes in CCI-induced allodynia, we found that the expression of P2X_4_R was significantly increased on postoperative days 7 and 10. Our findings suggest that the upregulation of P2X_4_R may contribute to initiate neuropathic pain. Consistent with this idea, it has been reported that P2X_4_R, mainly expressed in microglia, is involved in the generation and maintenance of neuropathic pain [23]. In the present study, we found that early HBO treatment beginning on postoperative day 1, but not on postoperative days 6 and 11, inhibited the CCI-induced upregulation of P2X_4_R, further suggesting that P2X_4_R contributes to initiate neuropathic pain, and early inhibition of P2X_4_R by HBO treatment could prevent the upregulation of P2X_4_R and thus inhibit neuropathic pain. In addition, it has been reported that the expression of P2X_4_R in the spinal cord is strongly upregulated at 7 and 10 days after nerve injury[28, 29]. Furthermore, Tanga et al. have reported that peripheral nerve injury induced an early spinal microglial activation that continued to increase until postoperative day 14 and returned to almost normal levels by postoperative day 28 [30]. Consistent with these studies, we found that the expression of P2X_4_Rreturned to normal levels at 21 days after nerve injury.

The mechanisms by which P2X_4_R contributes to neuropathic pain are not well understood. It is known that in response to peripheral nerve injury, P2X_4_R is upregulated in activated microglia and activated by ATP released from damaged neurons. Activation of P2X_4_R in activated microglia can increase the release of excitatory glutamate and open voltage-gated sodium channels, reduce the release of inhibitory GABA and glycine, inactivate potassium channels, and inhibit K^+^/Cl^-^ cotransporters, eventually resulting in neuropathic pain [31]. In addition, it has been reported that the release of cytotoxic substances such as interleukin-6, tumor necrosis factor (TNF)-α, and nitric oxide from activated microglia in response to inflammatory mediators is associated with the generation and maintenance of neuropathic pain; moreover, reduction of the number of microglia and inhibition of microglial activation produce analgesia [32,33], suggesting that microglial activation may play an important role in neurological pain. Furthermore, it has been reported that the upregulation of P2X_4_R is involved in microglial activation, and it mediates the release of inflammatory mediators and neuropathic pain [34,35]. The released inflammatory mediators from activated microglia further damage neurons by promoting the release of neurotoxic cytokines and inflammatory mediators from the surrounding microglia and astrocytes, leading to inflammatory injury to neurons and aggravation of neuropathic pain [36]. Inhibition of microglial activation by specific inhibitors can effectively block neuropathic pain [32,33], further suggesting that microglial activation is important in neuropathic pain. Li et al. have reported that HBO treatment alleviated CCI-induced neuropathic pain, which was associated with the reduction of TNF production [20], indicating that HBO treatment may produce antinociceptive effects via suppression of inflammatory mediators. In the present study, we further demonstrated that early HBO treatment inhibited CCI-induced neuropathic pain and reduced the expression of P2X_4_R, suggesting that early HBO treatment may inhibit microglial activation via downregulation of P2X_4_R.

Apoptosis is known to be involved in neuropathic pain following spinal nerve injury [24]. Sugimoto et al.[37] have reported that chronic constriction injuryevoked apoptosis in spinal dorsal horn neurons and that apoptosis continued to increase up to 14 days. Similarly, we found that CCI induced cell apoptosis on postoperative day 7 and that apoptosis continued to increase up to 21 days. Consistent with the changes in apoptosis, we found that MWT and TWL were significantly decreased on postoperative day 7 and were maintained for 14 days. These findings suggest that cell apoptosis may contribute to the persistent neuropathic pain following spinal nerve injury. Caspase 3, a member of the cysteine-aspartic acid proteinase (caspase) family, is well known to be involved in cell apoptosis [38,39] and has been found to play an important role in neuropathic pain in rats [24,39]. In the present study, we found that CCI induced an increase in the expression of caspase 3, which is associated with nuclear fragmentation and lysis of the neuronal ultrastructure, further confirming that caspase 3-mediated cell apoptosis may play a role in CCI-induced neuropathic pain. Furthermore, we found that late HBO treatment beginning on postoperative day 11, but not on postoperative days 1 and 6, produced persistent antinociceptive effects and reduced CCI-induced cell apoptosis, suggesting that the antinociceptive mechanism of late HBO treatment is due to inhibition of cell apoptosis. Furthermore, late HBO treatment inhibited the CCI-induced increase in the expression of caspase 3 and improved ultrastructural damage, further indicating that HBO treatment may alleviate neuropathic pain via caspase 3-mediated apoptosis.

Although we investigated the antinociceptive effect of HBO treatment using several methods, including immunohistochemistry, western blot analysis, RT-PCR, and TUNEL staining, the sample size (n = 2 for each group) is small. The small sample size leads to a relatively broad variation in these biochemical assays and thus may reduce accurate evaluation of our data. Further studies with a larger sample size are required to confirm the findings of our study.

In summary, we investigated the antinociceptive effects of HBO treatment at various stages following CCI in rats. Early HBO treatment beginning on postoperative day 1 produced persistent antinociceptive effects and reduced the expression of P2X_4_R without changing CCI-induced apoptosis. In contrast, late HBO treatment beginning on postoperative day 11 produced persistent antinociceptive effects and inhibited CCI-induced apoptosis via downregulation of caspase 3 without changing the expression of P2X_4_R. These findings suggest that HBO treatment at various stages following CCI has different antinociceptive mechanisms: early HBO treatment is likely due to inhibition of P2X_4_R activation on the microglia, whereas late HBO treatment is likely due to inhibition of caspase 3-mediated apoptosis.
